# Genome‐Wide Comparative Analysis of WRKY Gene Family Explores Insight Into the Evolution and Expression Divergence in the Genus *Triticum*

**DOI:** 10.1002/fsn3.71160

**Published:** 2025-12-22

**Authors:** Xuanxuan Li, Xin Gao, Zhong Wang, Jia Shi, Hongzhi Zhang, Lihong Wang, Jianqiang Xia, Chunsheng Wang, Zhun Zhao, Yueqiang Zhang, Jianfeng Li

**Affiliations:** ^1^ Xinjiang Crop Chemical Regulation Engineering Technology Research Center, Key Laboratory of Northwest Oasis Water‐Saving Agriculture of Ministry of Agriculture and Rural Affairs, Key Laboratory of Desert Oasis Crop Physiology, Ecology and Tillage of Ministry of Agriculture and Rural Affairs Crop Research Institute of Xinjiang Academy of Agricultural Sciences Xinjiang China; ^2^ Xinjiang Crop Biotechnology Key Laboratory, Xinjiang Uygur Autonomous Region Academy of Agricultural Sciences Biological Breeding Laboratory Xinjiang China

**Keywords:** expression divergence, hexaploidy, polyploidy, tandem duplication, tetraploid, *Triticum*, wheat

## Abstract

Wheat ( *Triticum aestivum* L.), a globally significant cereal crop, plays a crucial role in human nutrition and agricultural systems. Recent studies have extensively examined the role of transcription factors across various plant genomes. However, comprehensive comparative genomic analyses of the WRKY gene family across various diploid, tetraploid, and hexaploid wheat genomes remain limited. Here, we conducted a detailed analysis of the WRKY gene family in one diploid, two tetraploid, and five hexaploid wheat genomes, identifying a total of 1912 WRKY genes. Consistent with previous reports, the genes were classified into three groups according to their conserved domain and motif architectures. Phylogenetic reconstruction resolved clear clades, with Group II forming the largest and most diverse branch. Tandem duplication events were found to significantly impact the expansion of the WRKY gene family, particularly in the *T. aestivum* acc. Chinese Spring genome, which exhibited the highest number of tandemly duplicated genes. Analysis of A subgenomes in polyploid wheat genomes suggested potential gene loss following polyploid formation. Expression profiling of WRKY genes in bread wheat under salt stress and autophagy inhibition conditions uncovered differential expression patterns and significant divergence among tandemly duplicated genes. This study provides valuable insights into the distribution, phylogenetic relationships, and expression patterns of WRKY genes in wheat. Our findings highlight the role of tandem duplication in driving functional divergence, offering important genetic resources for wheat gene editing and genome‐assisted breeding.

## Graphical abstract

Our manuscript “Genome‐wide comparative analysis of WRKY gene family explores insight into the evolution and expression divergence in the genus Triticum” has been returned to draft so you can make corrections. In order for your manuscript to be considered by Food Science and Nutrition, please address the following items:

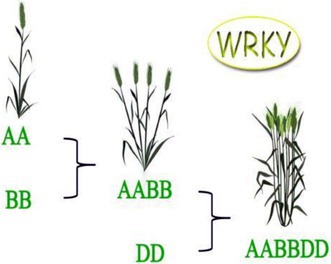

Additional information is required before this submission can be considered further.

Additional comments: The Graphical abstract should be provided in a Word file format with the following information: (a) Article title. Response: The analysis of WRKY gene family in the genus Triticum. (b) Authors' names (with the corresponding author indicated by an asterisk). Response: We have checked all the authors' names in the manuscript. The names of all authors are as follows: Xuanxuan Li, Xin Gao, Zhong Wang, Jia Shi, Hongzhi Zhang, Lihong Wang, Jianqiang Xia, Chunsheng Wang, Zhun Zhao, Yueqiang Zhang, Jianfeng Li. (c) No more than 80 words or 3 sentences of text summarizing the key findings presented in the paper. Response: Comparative WRKY analysis across nine wheat genomes reveals 1912 genes, tandem duplication‐driven expansion, and stress‐responsive divergence for breeding. (d.) A figure that best represents the scope of the paper. The image supplied should fit within the dimensions of 50 mm x 60 mm, and be fully legible at this size.

## 1 Introduction

Wheat ( *Triticum aestivum* L.) is a globally significant cereal crop that plays a crucial role in human nutrition and agricultural systems. It occupies about 17% of the world's total crop acreage, serving as a staple for nearly 40% of the global population and providing 20% of the total dietary calories and protein intake (Acevedo et al. 2018). Adapted to temperate climates, wheat has a long domestication history, dating back approximately 10,000 years, making it one of the earliest domesticated crops (Katamadze et al. 2023). Common wheat has a complex hexaploid genome (AABBDD) with three subgenomes (A, B and D), each containing 7 chromosomes (haploid *n* = 21) (Tiwari et al. 2024). The genome originated through two major allopolyploidization events: the formation of tetraploid wild emmer wheat ( *T. turgidum* ssp. *dicoccoides*, AABB) from the hybridization of *T. urartu* (AA) and a species related to *Aegilops speltoides* (BB), and the formation of hexaploid wheat from the hybridization of tetraploid wild emmer wheat with *A. tauschii* (DD) (Wang et al. 2024).

To identify genes or variations associated with key traits or phenotypes in wheat, a diverse range of wheat accessions has been finished with genome sequencing. For the A subgenome, the genome of *T. urartu* (AA) accession G1812 serves as a crucial foundation for genetic analysis (Ling et al. 2018). In tetraploid wheat (AABB), sequenced accessions such as durum wheat ( *T. turgidum ssp. durum* ) Svevo and wild emmer wheat ( *T. turgidum* ssp. *dicoccoides*) Zavitan offer valuable insights into genetic diversity and evolutionary pathways (Avni et al. 2017). For hexaploid wheat (AABBDD), several cultivars, including “Chinese Spring,” “Fielder,” “Kariega,” “KN9204,” and “Renan,” have had their genomes sequenced (Liu et al. 2024). Among them, “Chinese Spring” is the reference genome, while cultivars like “Renan” provide unique genetic traits that contribute to a more comprehensive understanding of the hexaploid wheat genome. The genome sequences of various wheat accessions with different karyotypes offer a genome‐wide data source for studying crucial gene families in wheat genomes.

The WRKY gene family, a crucial component of the plant transcriptional regulatory system, is characterized by a conserved WRKY domain containing the sequence WRKYGQK and a zinc‐finger motif, enabling classification into three main groups. Group I contains two WRKY domains and one C2H2‐type zinc finger structure, with the WRKY domains located at both the N‐terminus and C‐terminus. Group II contains one WRKY domain and one C2H2‐type zinc finger structure, and Group III contains one WRKY domain and two different types of zinc finger structures (C2H/C and C2H2) (Rushton et al. 2010). Genomic analysis reveals that the number of WRKY genes varies among plants; for example, *Arabidopsis thaliana* has about 74, rice approximately 109, and soybean over 180, which is attributed to evolutionary divergence and ecological adaptations (Yu et al. 2016). Functionally, they are involved in multiple plant processes. *AtWRKY6* in Arabidopsis affects seed germination through hormone‐related genes (Tang et al. 2023), and *OsWRKY13* in rice influences root development (John et al. 2019). In stress responses, *PtoWRKY13* in tomato activates PR genes against *Pseudomonas syringae* (Wang et al. 2012), *TaWRKY1* in wheat defends against *Puccinia striiformis* (Asghar et al. 2025), and *CsWRKY40* in cucumber responds to cucumber mosaic virus (Jacquemond 2012). In abiotic stress, especially salt stress, *AtWRKY25* and *AtWRKY33* in Arabidopsis are highly induced and regulate genes like *SOS1* for ion homeostasis to enhance salt tolerance (Jiang and Deyholos 2009). Additionally, *ZmWRKY53* in maize aids in drought tolerance (Pak et al. 2024), *AtWRKY45* in Arabidopsis deals with salt stress (Zhou et al. 2025), and *PtWRKY23* in poplar is for cold stress (Levée et al. 2009).

Here, the investigation undertook an exhaustive genome‐wide comparative analysis of the WRKY gene family across eight wheat genomes, encompassing one diploid, two tetraploid, and one hexaploid wheat genome. We identified the members of the WRKY gene family and classified them into three groups based on their domain and motif characteristics in eight wheat genomes. Phylogenetic analysis revealed distinct clustering patterns, with Group II emerging as the largest in eight wheat genomes. By conducting evolutionary analyses, we elucidated the genesis and evolutionary trajectories of the WRKY gene family within the A subgenome of wheat. Moreover, we assessed the influence of tandem duplication (TD) events on the emergence of WRKY genes through a comprehensive genome‐wide TD analysis. Additionally, we examined the functional divergence of WRKY genes under varying salt stress conditions via transcriptomic profiling in *T. aestivum* (AABBDD, Chinese Spring). Collectively, these results provide novel insights into the evolutionary dynamics and expression profiles of the WRKY gene family, thereby establishing a foundation for genetic breeding and molecular improvement strategies in diploid, tetraploid, and hexaploid wheat species.

## 2 Materials and Methods

### 2.1 Data Collection

Genomic data of the progenitor of wheat A subgenome *T. urartu* (diploid, AA) acc. G1812, wild tetraploid wheat, durum wheat, *Triticum turgidum ssp. durum* (tetraploid, AABB) acc. Svevo, wild emmer wheat, *T. turgidum* ssp. *dicoccoides* (tetraploid, AABB) acc. Zavitan, bread wheat, *T. aestivum* (hexaploid, AABBDD) acc. Chinese Spring, Fielder, Kariega, KN9204, and Renan were downloaded from NCBI Genomes (https://www.ncbi.nlm.nih.gov/datasets/genome/). Genomic data of Arabidopsis version Araport11 were downloaded from TAIR (https://www.arabidopsis.org/) (Cheng et al. 2017). The HMM profile of WRKY DNA ‐binding domain (PF03106.21) was downloaded from Pfam 37.1 (http://pfam‐legacy.xfam.org/) (Mistry et al. 2021). The RNA‐seq data of root and leaf under the processes of different NaCl concentrations from bread wheat, *Triticum aestivum* (hexaploid, AABBDD) cv. Jimai 22 were downloaded from the Sequence Read Archive (SRA) database with accession numbers: PRJNA699868 (Yue et al. 2021).

### 2.2 The Retrieval of WRKY Transcription Factors

All the candidate WRKY proteins were retrieved using the implementation of HMMER (v3.2.1‐foss‐2018b) with “trusted cutoff” as the threshold from eight wheat genomes (Potter et al. 2018). For the WRKY proteins from the same loci on the wheat chromosome, the longest WRKY proteins were retained to be served as the representatives. In each wheat genome, the filtered candidate WRKY protein sequences with highly conserved WRKY domain (PF03106.21) were used to perform multiple sequence alignments (MSA) using MAFFT v7.520 software (Katoh et al. 2019). Further, HMMER (v3.2.1‐foss‐2018b) was employed to build a species‐specific HMM profile of the WRKY domain and then identify the WRKY proteins in eight wheat diploid, tetraploid, and hexaploid genomes. The final WRKY proteins were validated with InterProScan version 5.67–99.0 across the wild diploid, wild tetraploid, and hexaploid bread wheat genomes (Jones et al. 2014). To investigate the WRKY groups, the MEME suite was employed to confirm the motifs in the protein sequences of WRKY across the wheat genomes (Bailey et al. 2015).

### 2.3 Phylogenetic Analyses of WRKY Transcription Factors

All the final WRKY proteins from eight wheat genomes were used to perform the MSA analyses using MAFFT v7.520 with the default parameters (Katoh et al. 2019). The WRKY multiple sequence alignment (MSA) was employed to construct phylogenetic trees using the maximum likelihood method. The analysis was performed with the IQ‐Tree v2.3.1 software, utilizing the following parameters: ‐bb 1000 ‐redo ‐alrt 1000 ‐m MFP ‐nt AUTO (Minh et al. 2020). For the comparison between Arabidopsis and wheat genomes, all the WRKY proteins from Arabidopsis and wheat genomes were merged and the phylogenetic analysis of WRKY proteins with Arabidopsis and wheat genomes was implemented to follow the above pipeline of the entire WRKY proteins from eight wheat genomes.

### 2.4 The Identification of Tandemly Duplicated WRKY Genes

Tandemly duplicated genes are the clustered genes that are similar or identical in sequence, arranged head‐to‐tail on the same chromosome, often sharing similar functions and likely arising through gene duplication and evolution. In each wheat genome, the identification of paralogous gene pairs was performed using the Diamond v0.9.24.125 software through all‐against‐all protein sequence mapping, with the following parameters: ‐e 1e‐5 ‐outfmt 6 ‐more‐sensitive ‐threads 62 ‐quiet (Buchfink et al. 2015). Utilizing the information on gene locations across chromosomes or assembled scaffolds in wheat genomes, we identified all tandemly duplicated genes across eight wheat genomes. These collinear clusters of tandemly duplicated genes were designated as tandem arrays.

### 2.5 The Identification of Collinear WRKY Gene Pairs

Orthologous gene pairs in the A subgenomes from tetraploid and hexaploid wheat genomes compared to diploid wheat *T. urartu* acc. G1812 genome were identified using MCScanX with the parameters: MATCH_SIZE = 5 and E_VALUE = 1e‐10 (Wang et al. 2012). The orthologous WRKY gene pairs were retrieved from the entire dataset of orthologous gene pairs. Further, the collinear WRKY gene pairs were identified, which can be used to perform the retention or loss of WRKY genes in A subgenomes in tetraploid and hexaploid wheat genomes.

### 2.6 Expression Analysis of WRKY Genes in Hexaploid Bread Wheat

To conduct our analysis, we obtained RNA‐seq data from the Sequence Read Archive (SRA). The raw sequencing data from different samples were processed using Trimmomatic v0.39 (Bolger et al. 2014), to trim and clean the reads, removing low‐quality bases and adapter sequences. The cleaned reads were then mapped to the hexaploid wheat Chinese Spring reference genome using HISAT2 (version 2.2.1) (Kim et al. 2015). Gene expression levels were quantified using StringTie version 2.0.6 (Pertea et al. 2015), which calculated the fragments per kilobase of exon model per million mapped fragments (FPKM) for the mapped reads. The FPKM values were normalized using the log2 transformation to facilitate accurate comparisons across samples. Differential expression analysis of WRKY genes under various conditions at the same time points was performed using the DESeq2 package (Love et al. 2014), with the criteria of |log2(FC)| > 1 and P‐value ≤ 0.05 to identify significant expression differences. The expression profiles of the WRKY genes were visualized using the heatmap module from the R package, providing a clear representation of the expression patterns across different conditions.

## 3 Result

### 3.1 Identification of WRKY Genes Across Various Wheat Genomes

With the characteristics of the conserved domains of WRKY proteins, we identified the WRKY genes across one diploid, two tetraploid, and five hexaploid wheat genomes. After curation, a total of 1912 WRKY genes were retained across the eight wheat genomes, including 118 in *T. urartu* acc. G1812, 170 in *T. turgidum ssp. durum* (durum wheat) acc. Svevo, 198 in *T. turgidum* ssp. dicoccoides (wild emmer wheat) acc. Zavitan, 307 in *T. aestivum* acc. Chinese Spring, 302 in *T. aestivum* acc. Fielder, 267 in *T. aestivum* acc. Kariega, 264 in *T. aestivum* acc. KN9204, and 286 in *T. aestivum* acc. Renan (Table 1). Due to wheat being a polyploid species, the tetraploid (AABB) and hexaploid (AABBDD) wheat genomes should have two and three times the WRKY genes compared with the diploid wheat genome, meaning that 236 (2*118) and 354 (3*118) WRKY genes (Li et al. 2025). From comparisons of WRKY genes among allopolyploid wheat genomes, the members of WRKY genes indicated smaller numbers in the two tetraploid and five hexaploid wheat genomes than in the wild diploid wheat genome, which may indicate the increase of gene dosage leading to the loss of WRKY in the polyploid wheat genomes.

**TABLE 1:** Statistics of WRKY genes within various wheat genomes.

Organism name	Sample	Karyotype	Accession No.	Total genes	WRKY genes	Percentage (%)
*Triticum urartu*	G1812	diploid, AA	GCA_003073215.2	35,768	118	0.33
*Triticum turgidum ssp. durum*	Svevo	tetraploid, AABB	GCA_900231445.1	63,993	170	0.27
*Triticum turgidum ssp. Dicoccoides*	Zavitan	tetraploid, AABB	GCF_002162155.2	66,764	198	0.3
*Triticum aestivum*	Chinese Spring	hexaploid, AABBDD	GCA_018294505.1	103,785	307	0.3
*Triticum aestivum*	Fielder	hexaploid, AABBDD	GCA_907166925.1	120,708	302	0.25
*Triticum aestivum*	Kariega	hexaploid, AABBDD	GCA_910594105.1	116,576	267	0.23
*Triticum aestivum*	KN9204	hexaploid, AABBDD	GWHBJWI00000000	147,600	264	0.18
*Triticum aestivum*	Renan	hexaploid, AABBDD	GCA_937894285.1	109,549	286	0.26

### 3.2 Phylogeny of WRKY Genes Across Various Wheat Genomes

In eight wheat genomes, 212 WRKY genes containing two WRKY domains belonged to Group I, and 1035 WRKY genes containing one WRKY domain and one motif of C2H2‐type zinc finger, the same as the motif of Group I WRKY genes, belonged to Group II, with the rest of the WRKY genes belonging to Group III. Group II is the largest group among the WRKY groups in various wheat genomes (Table S1).

**FIGURE 1:**
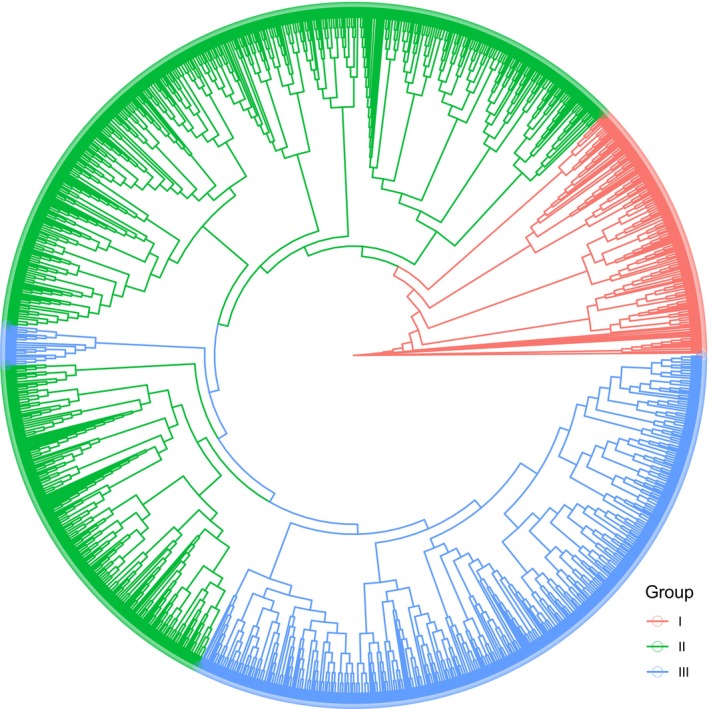
Phylogeny of WRKY genes across eight wheat genomes. The red, green, and blue colors represent the Group I, II, and III of WRKY gene family in wheat genomes.

To detect the phylogenetic relationship among different groups of the WRKY gene family, the entire WRKY protein sequences (1912) were used to construct the phylogenetic tree (Figure 1). The Group I genes within the WRKY family were clustered together, but the WRKY genes in Group III were clustered into two separate subgroups and one smaller group containing 26 WRKY genes (G1812: 2, Svevo: 3, Zavitan: 3, Chinese Spring: 6, Fielder: 6 Kariega: 2, and Renan: 4) was clustered within Group II of the WRKY gene family. Interestingly, all WRKY genes in wheat genomes are located on chromosomes 3 or 7 of the A, B, and D subgenomes, apart from two WRKY genes in the *T. urartu* acc. G1812 genome (Table S1).

### 3.3 Comparison of WRKY Genes in Arabidopsis and Various Wheat Genomes

**FIGURE 2:**
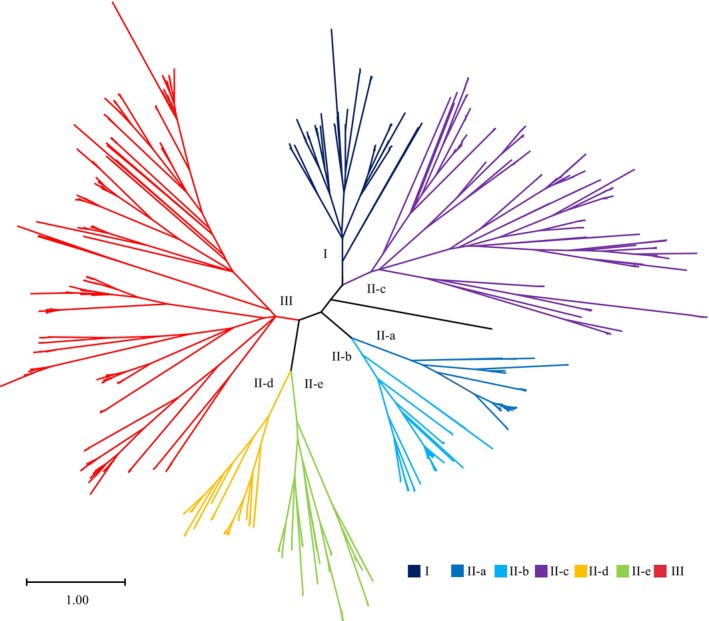
Phylogenetic tree of the WRKY gene family in *Arabidopsis* and wheat genomes. The (sub)groups of the WRKY gene family in *Arabidopsis* and wheat genomes are displayed in the no root phylogenetic tree with different colors.

Previous studies reported the WRKY genes in the Arabidopsis genome based on the previous version and classified the entire WRKY genes into three different groups according to the characteristics of conserved domains and motifs in WRKY protein sequences. Based on the HMMER search with HMM profiles, we identified 72 WRKY genes in the latest Arabidopsis genome TAIR11 (Cheng et al. 2017). Merged with the entire WRKY genes in eight wheat genomes, a total of 1984 WRKY protein sequences were used to perform the phylogeny analysis in Arabidopsis and various wheat genomes. All the WRKY genes were clustered into three major groups including Group I, II, and III. Group II was divided into II‐a, II‐b, II‐c, II‐d, and II‐e subgroups. Interestingly, in the Arabidopsis genome TAIR11, the WRKY10 gene (AT1G55600.1) was found to be separate yet closely related to both Group I and the II‐c subgroup. However, according to the classification criteria for WRKY proteins, the Arabidopsis WRKY10 was categorized as belonging to Group I (Figure 2).

Across Arabidopsis and eight wheat genomes, more than 50% of total WRKY genes were distributed into Group II of the WRKY gene family, and the II‐c subgroup contained the most WRKY genes among the subgroups in Group II (Table 2). *T. aestivum* acc. Kariega genome had the highest proportion and the biggest number of WRKY genes in Group II and the II‐c subgroup across the eight wheat genomes, respectively. However, the *T. aestivum* acc. Kariega genome had the lowest proportion of WRKY genes in Group III of the WRKY gene family within the eight wheat genomes, and the remaining seven wheat genomes exhibited a distribution of more than 32% of their total WRKY genes within Group III of the WRKY gene family. The *T. aestivum* acc. Kariega genome had the highest proportion of WRKY genes in Group I of the WRKY gene family across the eight wheat genomes. The proportion of WRKY genes in Group I from the Arabidopsis genome was higher than those in all the analyzed wheat genomes.

**TABLE 2:** Statistics of the groups of WRKY gene family.

Karyotype	Accession	Group I	Percentage Group I,%	Group II					Percentage Group II,%	Group III	Percentage Group III,%	Total
				II‐a	II‐b	II‐c	II‐d	II‐e				
Diploid, AA	G1812	11	9.32	7	12	32	9	5	55.08	42	35.59	118
Tetraploid, AABB	Svevo	21	12.35	11	22	37	14	9	54.71	56	32.94	170
	Zavitan	21	10.61	15	19	45	16	10	53.03	72	36.36	198
Hexaploid, AABBDD	Chinese Spring	30	9.77	24	33	67	23	14	52.44	116	37.79	307
	Fielder	33	10.93	22	33	66	22	13	51.66	113	37.42	302
	Kariega	33	12.36	21	36	68	24	15	61.42	70	26.22	267
	KN9204	30	11.36	19	31	56	20	13	52.65	95	35.98	264
	Renan	33	11.54	20	31	64	22	15	53.15	101	35.31	286
Arabidopsis TAIR11		12	16.67	3	10	20	7	4	61.11	16	22.22	72

### 3.4 Analysis of Tandem Duplication Events for WRKY Gene Family Across Eight Wheat Genomes

Tandem duplication plays a crucial role in plants by increasing the number of gene family members, which in turn expands the gene family (Li et al. 2025). The impact of tandem duplication events on the expansion of the WRKY gene family was investigated across eight wheat genomes. A total of 24, 14, 38, 66, 63, 52, 49, and 55 tandemly duplicated genes in *T. urartu* acc. G1812, *T. turgidum ssp. durum* acc. Svevo, *T. turgidum* ssp. *dicoccoides* acc. Zavitan, *T. aestivum* acc. Chinese Spring, Fielder, Kariega, KN9204, and Renan, representing 20.34%, 8.24%, 19.19%, 21.5%, 20.86%, 19.48%, 18.56%, and 19.23% of the entire WRKY genes in the corresponding wheat genomes, respectively (Table 3). Noteworthily, *T. urartu* acc. G1812, the progenitor of wheat A subgenome from tetraploid and hexaploid wheat, had more tandemly duplicated WRKY genes than those in the tetraploid wheat *T. turgidum ssp. durum* acc. Svevo and *T. turgidum* ssp. *dicoccoides* acc. Zavitan, indicating that the *T. urartu* acc. G1812 experienced independent tandem duplication events after the formation of tetraploid wheat genomes. Out of the eight wheat genomes, tetraploid wheat *T. turgidum ssp. durum* acc. Svevo and hexaploid wheat *T. aestivum* acc. Chinese Spring had the lowest and highest proportion of tandemly duplicated WRKY genes, indicating the weakest and strongest influence of tandem duplication events on the WRKY gene family, respectively. Among the eight wheat genomes, *T. turgidum ssp. durum* acc. Svevo possessed the fewest tandemly duplicated WRKY genes, with a total of 14 genes distributed across seven two‐gene tandem arrays. In contrast, the *T. aestivum* acc. Chinese Spring genome had the highest number of tandemly duplicated WRKY genes, comprising 66 genes that were distributed into 28 tandem arrays, including 19 two‐gene, eight three‐gene, and one four‐gene tandem arrays (Supplemental Table S2).

**TABLE 3:** Tandem duplication events of the members of the WRKY gene family.

Organism Name	Sample	Karyotype	WRKY genes	TD WRKY genes	Percentage (%)	No. tandem array
*Triticum urartu*	G1812	diploid, AA	118	24	20.34	11
*Triticum turgidum ssp. durum*	Svevo	tetraploid, AABB	170	14	8.24	7
*Triticum turgidum ssp. Dicoccoides*	Zavitan	tetraploid, AABB	198	38	19.19	17
*Triticum aestivum*	Chinese Spring	hexaploid, AABBDD	307	66	21.5	28
*Triticum aestivum*	Fielder	hexaploid, AABBDD	302	63	20.86	27
*Triticum aestivum*	Kariega	hexaploid, AABBDD	267	52	19.48	21
*Triticum aestivum*	KN9204	hexaploid, AABBDD	264	49	18.56	22
*Triticum aestivum*	Renan	hexaploid, AABBDD	286	55	19.23	25

### 3.5 Retention or Loss of WRKY Genes in the A Subgenomes in Tetraploid and Hexaploid Wheat Genomes

The analysis of eight wheat genomes, comprising one diploid, two tetraploid, and five hexaploid genomes, revealed a distinct distribution of WRKY genes. In the polyploid species, these genes were allocated across the A, B, and D subgenomes. Comparative analysis showed that the B subgenome harbored fewer WRKY genes than the A subgenome in tetraploid wheat and the A and D subgenomes in hexaploid wheat (Table 4). Notably, the A subgenome in both tetraploid and hexaploid wheat consistently exhibited a lower number of WRKY genes compared to the diploid wheat genome, suggesting a potential loss of WRKY genes in the A subgenome following the formation of polyploid wheat genomes. Furthermore, the A subgenome of the tetraploid wheat *T. turgidum ssp. durum* acc. Svevo contained the fewest WRKY genes among all polyploid wheat A subgenomes.

**TABLE 4:** Summary of WRKY genes in the A, B, and D subgenomes in tetraploid and hexaploid wheat genomes.

Organism name	Samples	Total WRKY genes	No. WRKY genes in subgenomes		
			AA	BB	DD
*Triticum urartu*	G1812	118	118	/	/
*Triticum turgidum ssp. durum*	Svevo	170	86	84	/
*Triticum turgidum ssp. Dicoccoides*	Zavitan	198	103	95	/
*Triticum aestivum*	Chinese Spring	307	105	92	110
*Triticum aestivum*	Fielder	302	105	90	107
*Triticum aestivum*	Kariega	267	89	85	93
*Triticum aestivum*	KN9204	264	89	83	92
*Triticum aestivum*	Renan	286	93	84	109

Hexaploid wheat originated from two major allopolyploidization events, with *T. urartu* serving as the progenitor of the A subgenome in both tetraploid and hexaploid wheat genomes. In this section, the *T. urartu* acc. G1812 genome was used as a reference to identify orthologous genes in the A subgenome of tetraploid and hexaploid wheat genomes. Subsequently, we investigated the retention or loss of WRKY genes in the A subgenomes of these wheat genomes. Compared with 118 WRKY genes in *T. urartu* acc. G1812 genome, a total of 46 and 62 orthologous WRKY genes were identified in the A subgenomes of tetraploid wheat *T. turgidum ssp. durum* acc. Svevo and *T. turgidum* ssp. *dicoccoides* acc. Zavitan, accounting for 38.98% and 52.54% of the total WRKY genes (118) in *T. urartu* acc. G1812 genome, respectively (Table 5). So, in the tetraploid genomes, the *T. turgidum* ssp. *dicoccoides* acc. Zavitan genome retained more ancestral WRKY genes from the progenitor of the A genome than the *T. turgidum ssp. durum* acc. Svevo after the formation of tetraploid wheat genomes. In hexaploid wheat genomes, a total of 66, 68, 17, 58, and 63 orthologous WRKY genes were detected in the A subgenomes from *T. aestivum* acc. Chinese Spring, Fielder, Kariega, KN9204, and Renan genomes, accounting for 55.93%, 57.63%, 14.41%, 49.15%, and 53.39% of the total WRKY genes (118) in *T. urartu* acc. G1812 genome, respectively. Among the five hexaploid wheat genomes, the *T. aestivum* acc. Fielder genome preserved the highest number of ancestral WRKY genes inherited from the A genome progenitor. In contrast, the *T. aestivum* acc. Kariega exhibited the opposite trend. These results indicated that the A subgenomes in tetraploid and hexaploid wheat genomes lost the most ancestral WRKY genes from the progenitors of the A subgenome after the species formation and further experienced independent evolution separately.

**TABLE 5:** The orthologous genes of the members of the WRKY gene family.

Organism Name	Samples	Total WRKY genes	No. orthologous WRKYs	Percentage (%), compared with G1812 WRKYs
*Triticum urartu*	G1812	118	0	0
*Triticum turgidum ssp. Durum*	Svevo	170	46	38.98
*Triticum turgidum ssp. Dicoccoides*	Zavitan	198	62	52.54
*Triticum aestivum*	Chinese Spring	307	66	55.93
*Triticum aestivum*	Fielder	302	68	57.63
*Triticum aestivum*	Kariega	267	17	14.41
*Triticum aestivum*	KN9204	264	58	49.15
*Triticum aestivum*	Renan	286	63	53.39

### 3.6 Expression Profile of WRKY Genes in Bread Wheat

To elucidate the expression profiles of WRKY genes in bread wheat, RNA‐seq data were obtained from bread wheat seedlings (roots and leaves) subjected to two treatments: 150 mM NaCl and 150 mM NaCl in combination with the autophagy inhibitor 3‐methyladenine (3‐MA) (Yue et al. 2021). This experiment aimed to examine the expression patterns of WRKY genes under both salt stress alone and salt stress with the added autophagy inhibitor 3‐MA. Including the control samples, a total of six distinct samples were generated, allowing for a comprehensive comparative analysis of the expression patterns of WRKY genes in bread wheat. Using an in‐house pipeline for RNA‐seq data analysis, it was found that 290 out of 307 WRKY genes were expressed across the six samples (Table S3). Among the total expressed WRKY genes, 286 were detected in the roots, while 256 were detected in the leaves. Specifically, 34 WRKY genes were uniquely expressed in the roots, and four WRKY genes were uniquely expressed in the leaves.

To elucidate the expression patterns of WRKY genes in bread wheat, their expression levels were normalized according to the measured values across six samples. Analysis revealed that the six samples were distinctly clustered according to tissue type. Moreover, all expressed WRKY genes were classified into two distinct expression clusters: Cluster I and II (Figure Figure S1). In Cluster I, eight WRKY genes showed high expression in bread wheat leaves under the condition of salt stress with the added autophagy inhibitor 3,‐MA; 21 WRKY genes showed high expression in bread wheat roots under the condition of salt stress, with 38 WRKY genes showing high expression in the roots under the condition of salt stress. In Cluster II, the expression of 55 WRKY genes in the control condition of bread wheat roots was significantly inhibited under different salt stresses, and 20WRKY genes showed high expression in the leaves under the condition of salt stress.

### 3.7 Expression Divergence of Tandemly Duplicated WRKY Genes

To investigate the evolutionary impact of tandem duplication (TD) events on the expression of WRKY genes in bread wheat, we analyzed the expression patterns of WRKY genes involved in TD events. Using the bread wheat Chinese Spring genome as the reference, we retrieved the expression values of these WRKY genes. In our analysis across eight wheat genomes, we identified 66 tandemly duplicated WRKY genes in the Chinese Spring genome. However, only 62 of these tandemly duplicated WRKY genes were found to be expressed in the seedlings of bread wheat, specifically in the roots and leaves under both control and treatment conditions. Out of 62 tandemly duplicated WRKY genes, four WRKY genes distributed into one four‐gene tandem array, 24 WRKY genes distributed into eight three‐gene tandem arrays, and 34 WRKY genes distributed into 17 two‐gene tandem arrays. Tandem arrays featuring identical gene copy numbers were positioned together to elucidate the differential expression patterns of tandemly duplicated WRKY genes.

**FIGURE 3:**
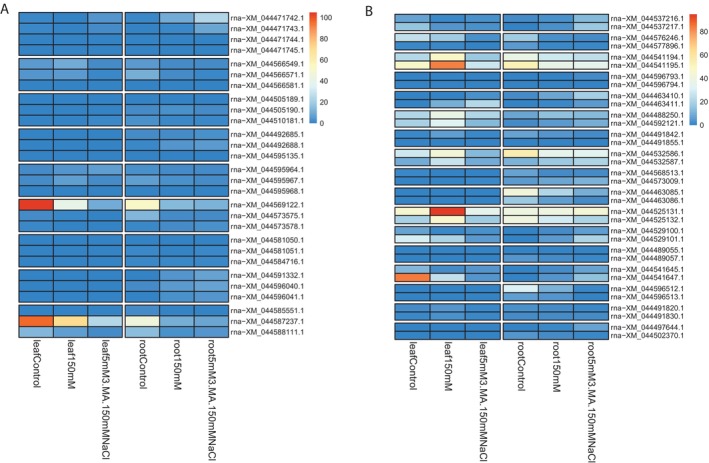
Expression profile of the tandemly duplicated *WRKY* genes. (A) Expression of *WRKY* genes from the four‐ and three‐gene tandem arrays; (B) expression of *WRKY* genes from two‐gene tandem arrays.

Through the analysis of expression, most tandemly duplicated WRKY genes have low expression values in roots or leaves under different conditions. In the three‐gene tandem array (*rna − XM_044569122.1*, *rna − XM_044573575.1*, and *rna − XM_044573578.1*), one member (*rna − XM_044569122.1*) exhibited high expression in leaves under control conditions. This suggests that its expression was inhibited under salt stress and salt stress with the added autophagy inhibitor 3‐MA. In contrast, the other two members of this array, along with another gene (*rna − XM_044587237.1*) in a different three‐gene tandem array (*rna − XM_044585551.1*, *rna − XM_044587237.1*, and *rna − XM_044588111.1*), consistently showed low expression across conditions (Figure 3A). In the two‐gene tandem array (*rna − XM_044525131.1* and *rna − XM_044525132.1*), one member displayed high expression in leaves under salt stress but low expression when the autophagy inhibitor 3‐MA was added (Figure 3B). Notably, both genes exhibited relatively high expression in roots under all conditions. Another two‐gene tandem array (*rna − XM_044541645.1* and *rna − XM_044541647.1*) showed similar expression patterns to the previously mentioned arrays, with one member (*rna − XM_044541647.1*) mirroring the expression divergence observed, while the other member maintained relatively high expression. These findings indicate that tandemly duplicated WRKY genes exhibit expression divergence under different conditions. Both functional redundancy and sub‐functionalization appear to have occurred during their evolution. Tandem duplication events likely play a significant role in driving functional divergence among WRKY genes in the bread wheat Chinese Spring genome.

## 4 Discussion

Interestingly, the number of WRKY genes in polyploid wheat genomes (tetraploid and hexaploid) was smaller than expected based on gene dosage, suggesting potential gene loss following polyploidization. This finding indicates that the increase in gene dosage may lead to the loss of WRKY genes in polyploid wheat genomes. The WRKY gene family is divided into three main groups based on the number of WRKY domains and zinc finger structures. Group II is the largest, containing the majority of WRKY genes across all wheat genomes. Phylogenetic analysis shows that WRKY genes in Group I cluster together, while those in Group III form two separate subgroups. This classification highlights the diversity and complexity of the WRKY gene family in wheat. The distribution of WRKY genes on chromosomes 3 and 7 of the A, B, and D subgenomes suggests specific genomic locations that may be important for their function and regulation.

Tandem duplication plays a crucial role in the expansion and functional diversification of the WRKY gene family in wheat. Analysis across eight wheat genomes identified a total of 320 tandemly duplicated WRKY genes, representing a significant proportion of the total WRKY genes in each genome. The highest proportion of tandemly duplicated WRKY genes was found in hexaploid wheat Chinese Spring, indicating strong tandem duplication events in this variety. In contrast, tetraploid wheat Svevo had the lowest proportion, suggesting weaker tandem duplication influence. The expression analysis of WRKY genes in bread wheat under salt stress and autophagy inhibition conditions revealed significant expression divergence among tandemly duplicated genes. For example, in a three‐gene tandem array, one member showed high expression in leaves under control conditions but was inhibited under stress, while the other two members maintained low expression across conditions. This expression divergence suggests that both functional redundancy and sub‐functionalization have occurred during the evolution of tandemly duplicated WRKY genes. These findings highlight the importance of tandem duplication in driving functional diversification of WRKY genes, which may contribute to the adaptation and stress tolerance of wheat.

## 5 Conclusion

This study offers a comprehensive analysis of the WRKY gene family across eight wheat genomes, including one diploid, two tetraploid, and five hexaploid species. A total of 1912 WRKY genes were identified, revealing significant variations in their distribution across different ploidy levels. Phylogenetic analysis using 1912 WRKY protein sequences revealed distinct clustering patterns, with Group II being the largest. The II‐c subgroup contained the most WRKY genes among the subgroups in Group II. The *T. aestivum* acc. Kariega genome had the highest proportion and number of WRKY genes in Group II and the II‐c subgroup, while the *T. urartu* acc. G1812 genome had a higher proportion of WRKY genes in Group I compared to all analyzed wheat genomes. Tandem duplication events were found to play a crucial role in the expansion of the WRKY gene family. The proportion of tandemly duplicated WRKY genes varied across the eight wheat genomes, with *T. urartu* acc. G1812 having the highest proportion (20.34%) and *T. turgidum ssp. durum* acc. Svevo having the lowest (8.24%). The *T. aestivum* acc. Chinese Spring genome had the highest number of tandemly duplicated WRKY genes (66 genes), distributed into 28 tandem arrays. Analysis of the A subgenomes in tetraploid and hexaploid wheat genomes revealed a potential loss of WRKY genes following polyploid formation. The A subgenome of *T. turgidum ssp. durum* acc. Svevo contained the fewest WRKY genes among all polyploid wheat A subgenomes. In hexaploid wheat genomes, the *T. aestivum* acc. Fielder genome preserved the highest number of ancestral WRKY genes inherited from the A genome progenitor, while the *T. aestivum* acc. Kariega genome exhibited the opposite trend. Expression profiling of WRKY genes in bread wheat under salt stress and autophagy inhibition conditions showed differential expression patterns. The expressed WRKY genes were classified into two distinct expression clusters, with significant expression divergence observed among tandemly duplicated genes. This suggests that both functional redundancy and sub‐functionalization have occurred during the evolution of tandemly duplicated WRKY genes, highlighting the significant role of tandem duplication in driving functional divergence in the bread wheat Chinese Spring genome.

In conclusion, this study provides valuable insights into the distribution, phylogenetic relationships, and expression patterns of WRKY genes across diverse wheat genomes. The findings highlight the significant impact of tandem duplication on the expansion and functional divergence of the WRKY gene family and underscore the potential loss of WRKY genes in the A subgenomes of polyploid wheat species. These results contribute to a deeper understanding of the WRKY gene family's evolution and its implications for stress response mechanisms in wheat.

## Author Contributions

**X.L.:** performed the experiments, analyzed the data, wrote, original draft, and reviewed drafts of the paper. **X.G.:** collected wheat germplasm resources, wrote, original draft and reviewed drafts of the paper. **Z.W.:** conceived and designed the experiments, analyzed the data, arranged the figures and tables, and reviewed drafts of the paper. **J.S.:** analyzed the data and reviewed drafts of the paper. **H.Z.:** analyzed the data and reviewed drafts of the paper. **L.W.:** analyzed the data and reviewed drafts of the paper. **J.X.:** analyzed the data and reviewed drafts of the paper. **C.W.:** analyzed the data and reviewed drafts of the paper. **Z.Z.:** analyzed the data and reviewed drafts of the paper. **Y.Z.:** conceived and designed the experiments, wrote the paper, and modified the paper. **J.L.:** conceived and designed the experiments, wrote the paper, and modified the paper.

## Conflicts of Interest

The authors declare no conflicts of interest.

## Supporting information


**Figure S1:** Expression heatmap of the entire WRKY genes in bread wheat Chinese Spring.

**Table S1:** The distribution and groups of the members of WRKY gene family across the eight wheat genomes.
**Table S2:** List of tandem array of WRKY genes across various wheat genomes.
**Table S3:** Expression values of WRKY genes in bread wheat.

## Data Availability

The data used in this article were downloaded from the NCBI website, and the data accession number is PRJNA699868.
