# Supplementary material for: Genome‐Wide Comparative Analysis of WRKY Gene Family Explores Insight Into the Evolution and Expression Divergence in the Genus Triticum
Source: Food Sci Nutr. 2025 Dec 22;13(12):e71160. doi: 10.1002/fsn3.71160 (PMC12719918; doi:10.1002/fsn3.71160)
Supplement: Supplementary file 1 — Figure S1: Expression heatmap of the entire WRKY genes in bread wheat Chinese Spring. [file FSN3-13-e71160-s001.docx]

**Supplemental figure S1**

Expression heatmap of the entire WRKY genes in bread wheat Chinese Spring.
